# A platinum(IV)–artesunate complex triggers ferroptosis by boosting cytoplasmic and mitochondrial lipid peroxidation to enhance tumor immunotherapy

**DOI:** 10.1002/mco2.570

**Published:** 2024-05-20

**Authors:** Renming Fan, Aohua Deng, Ruizhuo Lin, Shuo Zhang, Caiyan Cheng, Junyan Zhuang, Yongrui Hai, Minggao Zhao, Le Yang, Gaofei Wei

**Affiliations:** ^1^ Institute of Medical Research Northwestern Polytechnical University Xi'an China; ^2^ Research & Development Institute of Northwestern Polytechnical University in Shenzhen Shenzhen China; ^3^ Precision Pharmacy & Drug Development Center Department of Pharmacy Tangdu Hospital Air Force Military Medical University Xi'an China

**Keywords:** artesunate, ferroptosis, lipid peroxidation, mitochondria, mitochondrial fission, mitochondrial fusion, tumor immunotherapy

## Abstract

Ferroptosis is an iron‐dependent cell death form that initiates lipid peroxidation (LPO) in tumors. In recent years, there has been growing interest on ferroptosis, but how to propel it forward translational medicine remains in mist. Although experimental ferroptosis inducers such as RSL3 and erastin have demonstrated bioactivity in vitro, the poor antitumor outcome in animal model limits their development. In this study, we reveal a novel ferroptosis inducer, oxaliplatin–artesunate (OART), which exhibits substantial bioactivity in vitro and vivo, and we verify its feasibility in cancer immunotherapy. For mechanism, OART induces cytoplasmic and mitochondrial LPO to promote tumor ferroptosis, via inhibiting glutathione‐mediated ferroptosis defense system, enhancing iron‐dependent Fenton reaction, and initiating mitochondrial LPO. The destroyed mitochondrial membrane potential, disturbed mitochondrial fusion and fission, as well as downregulation of dihydroorotate dehydrogenase mutually contribute to mitochondrial LPO. Consequently, OART enhances tumor immunogenicity by releasing damage associated molecular patterns and promoting antigen presenting cells maturation, thereby transforming tumor environment from immunosuppressive to immunosensitive. By establishing in vivo model of tumorigenesis and lung metastasis, we verified that OART improves the systematic immune response. In summary, OART has enormous clinical potential for ferroptosis‐based cancer therapy in translational medicine.

## Graphical abstract

Ferroptosis is an iron‐dependent cell death form. In this study, we reveal a novel platinum(IV)–artesunate complex, which exhibits substantial bioactivity. For mechanism, the complex catalyzes lipid peroxidation, which ultimately induces ferroptosis in tumor cells. Meanwhile, the complex destroys ferroptosis defense system in mitochondria and cytoplasm. In summary, the complex has enormous clinical potential for ferroptosis‐based cancer therapy in translational medicine.

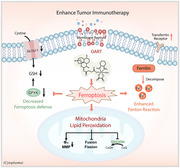

## 1 INTRODUCTION

Ferroptosis is an iron‐dependent cell death form that is driven by lipid peroxidation (LPO).^1^ Since concept of ferroptosis was proposed by Dr. Brent R. Stockwell for the first time in 2012,^2^ scientists have never stopped expanding its mechanism. The crux of ferroptosis execution is iron‐catalyzed LPO.^3^, ^4^ There are mainly three processes involved in ferroptosis: iron metabolism, lipid metabolism, and redox balance.^5^ For details, overloading iron, exogenous uptake or decomposition from ferritin, directly causes ferroptosis by accumulating labile iron pool,^1^ which is an important reactant of Fenton reaction.^6^ Besides, oxygen‐free radical catalyzes polyunsaturated fatty acid to produce cytotoxic lipid peroxides, which damage cells and rupture membranes, and this process is called LPO.^4^, ^7^, ^8^ Simultaneously, intrinsic reductive substance would be massively generated to counterbalance reduction system, including glutathione (GSH) and ubiquinol.^7^, ^9^, ^10^ GSH is mediated by glutathione peroxidase (GPX4), which is recognized as a classical antioxidant enzyme that detoxifies LPO.^11^, ^12^ Cystine/glutamate transporter Solute Carrier Family 7 Member 11 (SLC7A11) contributes to GSH synthesis via regulating amino acids transportation. Dihydroorotate dehydrogenase (DHODH) is an enzyme that promotes ubiquinol production, which is an emerging ferroptosis suppressor and mediate redox balance in mitochondria.^9^

In recent years, researchers have paid increasing attention on it, but how to propel ferroptosis forward translational medicine is still in mist. Experimental ferroptosis inducers such as GPX4 inhibitor RSL3 and SLC7A11 inhibitor erastin impose precise ferroptosis bomb on cancer cells in vitro. However, they tend to produce poor antitumor outcome in animal model, due to the comprehensive activation of ferroptosis on nontumor sites, such as dendrite cells (DCs) and T cells in immune organs.^13^, ^14^, ^15^, ^16^ Therefore, there is an urgent need to explore an efficient and tumor‐specific ferroptosis inducer that can be applied for cancer therapy.

Strategies for developing tumor‐specific agents based on ferroptosis has been developed; however, problems still exist. Iron‐based nanoparticles can solve the problem of endogenous iron deficiency,^17^, ^18^, ^19^, ^20^, ^21^ but they can also lead to turbulent disorders in iron metabolism, which raises biosafety concerns. Activating ferroptosis by photodynamical therapy or chemodynamical therapy is unable to maintain a continuous and sustainable process. Moreover, photodynamic therapy and chemodynamic therapy produce excess peroxides that damage immunocytes and lead to an immunosuppressive tumor environment.^22^, ^23^, ^24^, ^25^

In this work, we construct a novel ferroptosis‐inducer oxaliplatin–artesunate (OART) with extraordinary tumor regression effect and conquer the immunosuppressive traits of single ferroptosis inducers, by using platinum(IV) strategy to chemically combine ferroptosis‐inducer artesunate (ART) and oxaliplatin (Oxa). We not only elucidated the mechanism basis of OART on triggering ferroptosis in tumor cells, especially the impact on mitochondria, but also demonstrated its effectiveness on inducing immunogenic cell death (ICD) and enhancing immunotherapy in murine therapy.

## 2 RESULTS

### 2.1 OART inhibits tumor cell proliferation in vitro

**FIGURE 1:**
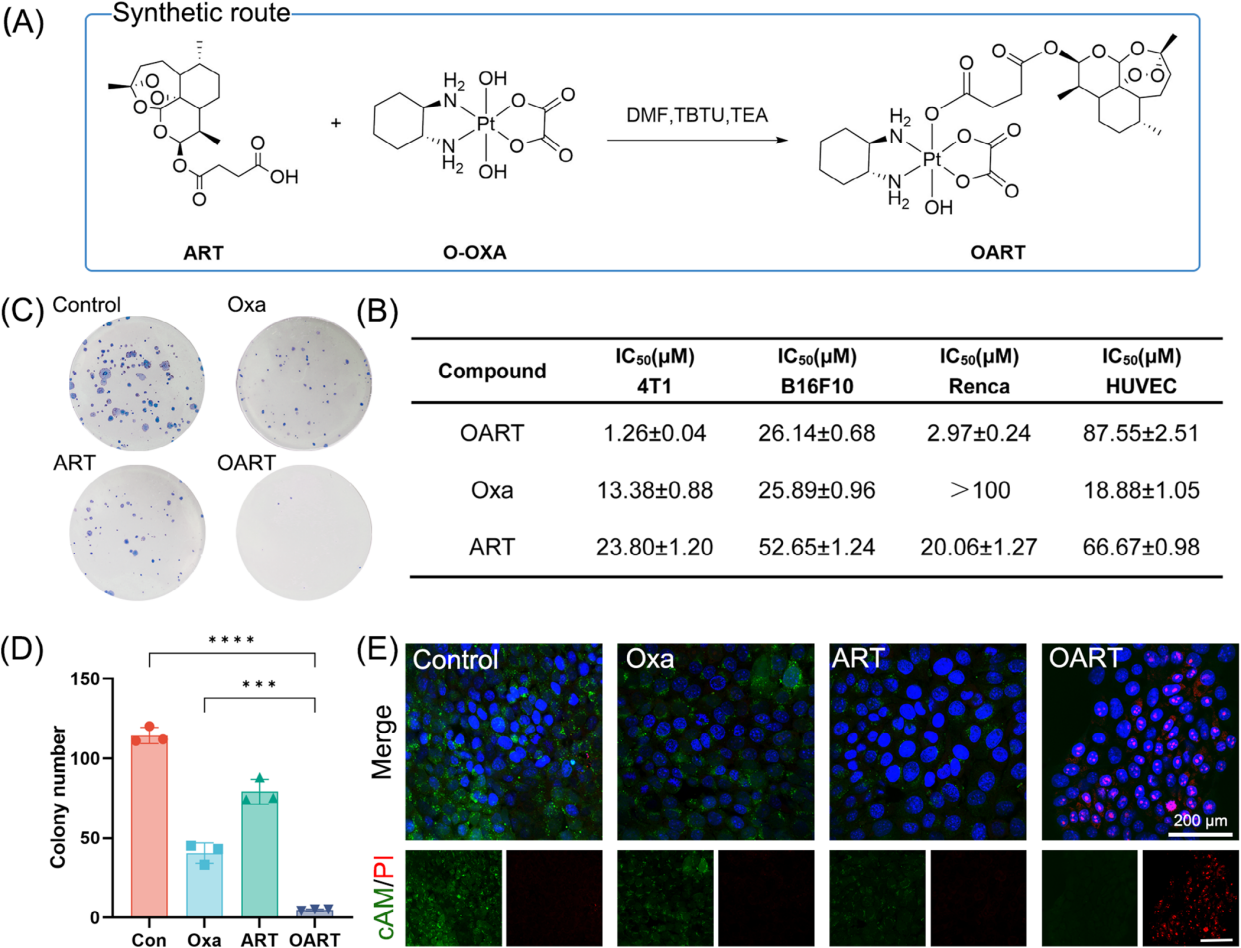
Evaluation on cytotoxicity of OART. (A) Synthetic method of platinum(IV) prodrug complexes OART. (B) IC_50_ value of OART, Oxa, and ART in multiple cell lines: 4T1, murine breast cancer cell lines; B16F10, murine melanoma cell lines; Renca, murine renal carcinoma; HUVEC, human umbilical vein endothelial cell (*n* = 6). (C and D) Colony formation assay on 4T1 cells (*n* = 3). (E) Living and dead cells stained with Calcein AM and PI. Scale bars = 200 μm. Data are expressed as mean ± SD. ***p < 0.001, ****p < 0.0001.

OART is a bifunctional molecule (Figure 1A) constructed from the ferroptosis‐inducer ART and the classical chemotherapeutics Oxa. To organically introduce Oxa into ART, we apply platinum(IV) strategy and design a new ferroptosis‐inducer agent OART through chemical synthesis instead of combinatory administration. Synthetic approach is presented in Figure 1A.^26^, ^27^, ^28^ The structure of OART was confirmed by ^1^H‐ NMR, ^13^C‐NMR, HRMS, and HPLC, as shown in Figures S1–S4.

The (3‐(4,5‐dimethylthiazol‐2‐yl)−2,5‐diphenyltetrazolium bromide) (MTT) assay reveals that OART exhibits extraordinary growth inhibition effect on multiple cancer cell lines, as shown in Figure 1B, which distinguishes it from both Oxa and ART. Among all experimental cells, murine breast cancer 4T1 cells are the most sensitive to OART with an IC_50_ value of 1.26 μM, while HUVEC cells are insensitive. Morphological observation shows that treatment with OART causes a time‐dependent damage on 4T1 cells (Figure S5). The colony formation assay reveals that OART dramatically deprives the proliferation ability of tumor cells in extreme low density (Figures 1C and D), indicating a wide destruction on tumorigenesis. A cell scratch assay was performed to determine cell migration ability, and we found cell migration rate in OART treatment group was much lower than control group (Figure S6). Calcein AM and propidium iodide (PI) were used to distinguish between living and dead cells. The gloomy green fluorescence suggests a deprivation of esterase activity in dead cells, whereas the red fluorescence emitted by PI indicates damage to DNA strand. The vanished green fluorescence and bright red fluorescence in OART group, as shown in Figure 1E, indicates a superior cytotoxicity on tumor cells compared with mono Oxa or ART. Besides, we have systematically evaluated apoptosis damage in 4T1 cells after treatment. As Figure S7 depicts, OART elevates the proportion of apoptotic cells by activating Caspase‐3 and upregulating γ‐H2AX. Overall, we synthesized OART bifunctional complex and verified its antitumor bioactivity in vitro.

### 2.2 OART induces ferroptosis in 4T1 cells

**FIGURE 2:**
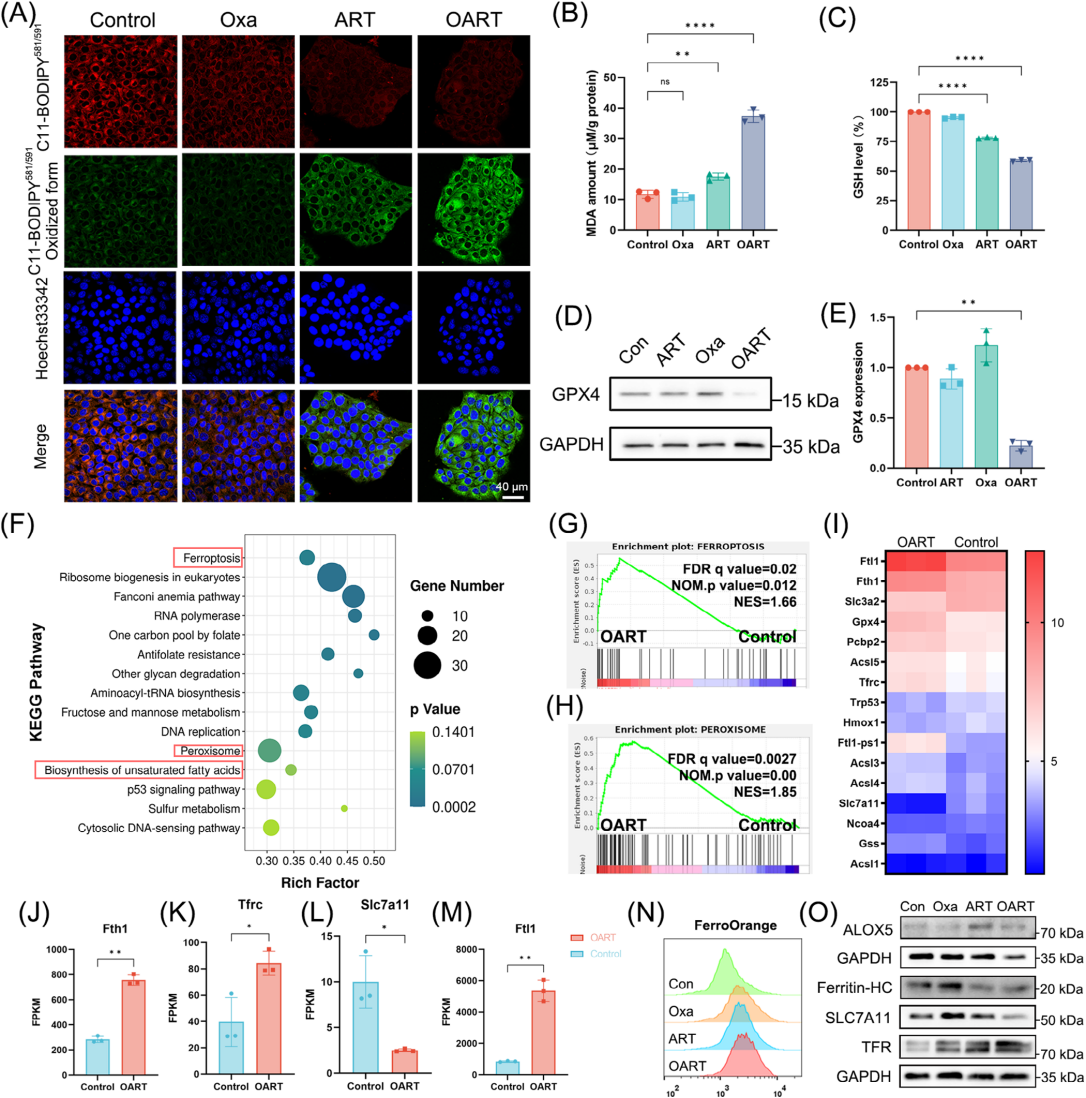
OART induces ferroptosis in 4T1 cells. (A) OART induces LPO in 4T1 cells as shown by upregulating oxidized form (green) and downregulating the reduced form (red) of C11‐Bodipy probe. Scale bars = 40 μm. (B and C) MDA and GSH content in 4T1 cells after treatment (*n* = 3). (D and E) GPX4 protein expression level in 4T1 cells after indicated treatment (*n* = 3). (F) KEGG enrichment in differential pathways (OART vs. control). (G and H) GSEA analysis shows that FERROPTOSIS and PEROXISOME pathways are upregulated in OART group. (I) Differential genes in FERROPTOSIS pathway (*n* = 3). (J–M) Relative abundance in genes including Fth1, Tfrc, Slc7a11, and Fthl1 (*n* = 3). (N) Intracellular ferrous iron content in 4T1 cells after treatment (*n* = 3). (O) Protein level on ALOX5, Ferritin HC, SLC7A11, TRF (*n* = 3). Data are expressed as mean ± SD. *p < 0.05, **p < 0.01, ***p < 0.001, ****p < 0.0001, ns, no significance.

Ferroptosis‐related indicators were evaluated in 4T1 cells following treatment. We primarily utilized a lipid probe C11‐Bodipy to detect the total LPO in whole cells and found it upregulated with OART treatment (Figure 2A). Consequently, an elevated levels of malondialdehyde (MDA), which is the terminal product of the process in LPO, suggested the complete LPO following treatment (Figure 2B). Meanwhile, we evaluated the integrity of defense system and results showed that intracellular GSH level in OART group was much lower than control (Figure 2C). In addition, the protein level of GPX4 showed a significant decrease (Figures 2D and E), which indicated a collapse on reduction capability to antiferroptosis system. Additionally, both blocking LPO by using Ferrostatin‐1 and chelating iron with deferoxamine can rescue OART‐induced cell death, which proves that OART acts as a ferroptosis‐dependent way to kill tumor (Figure S8).

Furthermore, we conducted RNA‐seq to comprehensively explore more details of OART's antitumor mechanism. Data were collected from four groups: DMSO, Oxa, ART, and OART. After examining the integrity of the output data, we analyzed the data and carried enrichment analysis using the Kyoto Encyclopedia of Genes and Genomes (KEGG) and Gene Ontology (GO). Gene Set Enrichment Analysis (GSEA) was also applied to select certain key pathway differentially expressed between treating and control group.

There was 2036 upregulated genes and 3368 downregulated genes between OART and control group (Figure S9). According to the KEGG enrichment plot, we noticed that ferroptosis and its related pathways, including peroxisome, sulfur metabolism, and biosynthesis of unsaturated fatty acid, inspiringly stood out with significant *p* value and high rich factor (Figure 2F). Furthermore, GSEA analysis revealed that genes in OART group were significantly enriched in FERROPTOSIS and PEROXISOME pathways (Figures 2G and H). While searching for the differential genes enriched in ferroptosis pathway, we collected data and plotted a heat map based on the fold change of genes (Figure 2I). Some typical ferroptosis‐related genes are differentially expressed including *Fth1, Tfrc, Slc7a11*, and *Fthl1* (Figures 2J–M). Intracellular ferrous iron concentration was evaluated by flow cytometry, and results showed that OART triggers iron accumulation in tumor cells (Figure 2N). Moreover, we determined protein content in cellular level and achieved great effect. Specifically, we found a downregulation of ferritin heavy chain (Ferritin‐HC) and an upregulation of transferrin receptor (TFR), as Figure 2O depicted, indicating an increase on extracellular iron uptake and intracellular ferritin degradation, ultimately leading accumulation on free iron. 5‐Lipoxygenase (ALOX5), a marker of LPO,^29^, ^30^ and SLC7A11, a membrane transporter for cysteine uptake, were also investigated. We found an increase of ALOX5 and decrease of SLC7A11 at protein level,^31^, ^32^ which contributed to ferroptosis. Herein, we conclude that OART induces ferroptosis via initiating LPO and depriving GSH, accompanied with a turbulence in ferrous homeostasis.

### 2.3 OART causes mitochondria damage and mitochondrial LPO

**FIGURE 3:**
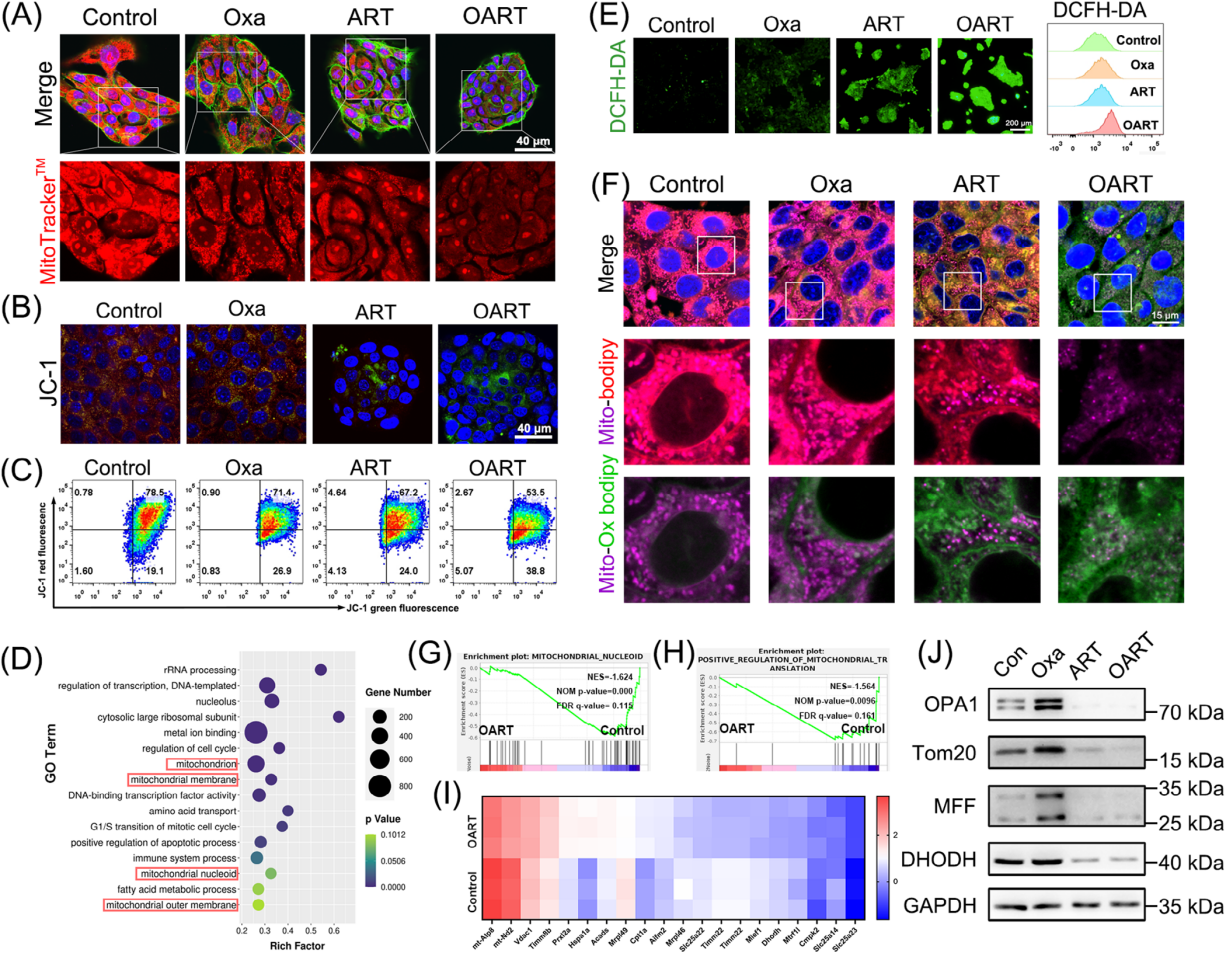
OART causes mitochondria damage and mitochondrial ferroptosis. (A) CLSM image of 4T1 cells tracked with MitoTracker™. Scale bars = 40 μm. (B) JC‐1 staining for 4T1 cells. Turnover of JC‐1 from red to green in 4T1 cells administrated with OART. Scale bars = 40 μm. (C) Flow cytometry determined MMP in 4T1 cells after indicated treatment (*n* = 3). (D) GO analysis on differential pathways (OART vs. control). (E) Intracellular ROS and lipid ROS were determined by CLSM and flow cytometry. Scale bars = 200 μm. (F) Colocalization of mitochondria and C11‐Bodipy probe. (G and H) GSEA analysis on mitochondrial nucleoid and translocation. Scale bars = 15 μm. (I) Heat map of genes related with mitochondria. (J) Protein expression on mitochondrial dynamics after indicated treatment (*n* = 3).

Mitochondrial damage is a characteristic feature of ferroptotic cells, we examined this using confocal laser scanning microscope (CLSM) and flow cytometry. As Figures 3A–C demonstrated, the dooming fluorescence on mito‐tracker and turnover on JC‐1 suggest that OART damages mitochondria and decreases mitochondrial membrane potential (MMP). Data from RNA‐seq demonstrated that pathways including mitochondria, mitochondrial nucleoid, and mitochondrial membrane are highly enriched and significantly changed in OART group (Figure 3D), which is consistent with the previous results.

Mitochondria are the key amplifiers of ferroptosis by generating lipid reactive oxygen (ROS) to catalyze the process of LPO. Intracellular ROS levels were detected by CLSM using DCFH‐DA, whereas lipid ROS in the cell membrane were captured by flow cytometry. Figure 3E shows that OART treatment increases fluorescence intensity, indicating that OART treatment damages mitochondria and triggers LPO by elevating ROS level. As Figure 3F depicted, the LPO probe C11‐Bodipy is predominately localized in mitochondria and the colocalization verified the occurrence on mitochondrial LPO.

Additionally, GSEA analysis revealed significant differences in pathways related to mitochondria fission and translocation between administrating group to control group (Figures 3G and H). Furthermore, a heatmap of 20 differentially expressed genes suggests that OART leads to mitochondrial damage at the transcriptional level (Figure 3I). However, the connection between mitochondrial damage and ferroptosis remains unclear.

Mitochondrial nucleoids are vital units containing mito‐DNA and proteins regulating mitochondrial fusion and fission,^33^ which is highly associated with mitochondrial dynamics.^34^ Thus, to gain further insight into the mechanism of mitochondrial damage, we determined the expression levels of proteins involved in mitochondrial dynamics. Optic Atrophy 1 (OPA1) is a key participant in mitochondrial fusion^35^, ^36^ and Tom 20 is a mitochondrial outer membrane receptor.^37^ The mitochondrial fission factor (MFF) is a key effector of mitochondrial fission.^37^ DHODH is a flavothin‐dependent enzyme containing iron and is located in the inner membrane of the mitochondria.^9^ Immunoblot results revealed that OPA1, Tom20, MFF, and DHODH were downregulated after ART and OART treatment (Figure 3J).

DHODH is reported to be a ferroptosis suppressor, independent of GPX4, which fights against LPO by producing panthenol. OART initiates mitochondrial lipid peroxidation by generating massive lipid ROS, disrupting DHODH defense system in the mitochondria. Combining these results with GPX–GSH system, we conclude that OART leads to double whammy on ferroptosis defense system. In contrast, OART inhibits mitochondrial fusion and fission, resulting in ROS leakage, and constant catalysis of LPO to induce tumor cell ferroptosis.

### 2.4 | OART inhibits 4T1 cell growth in vivo

**FIGURE 4:**
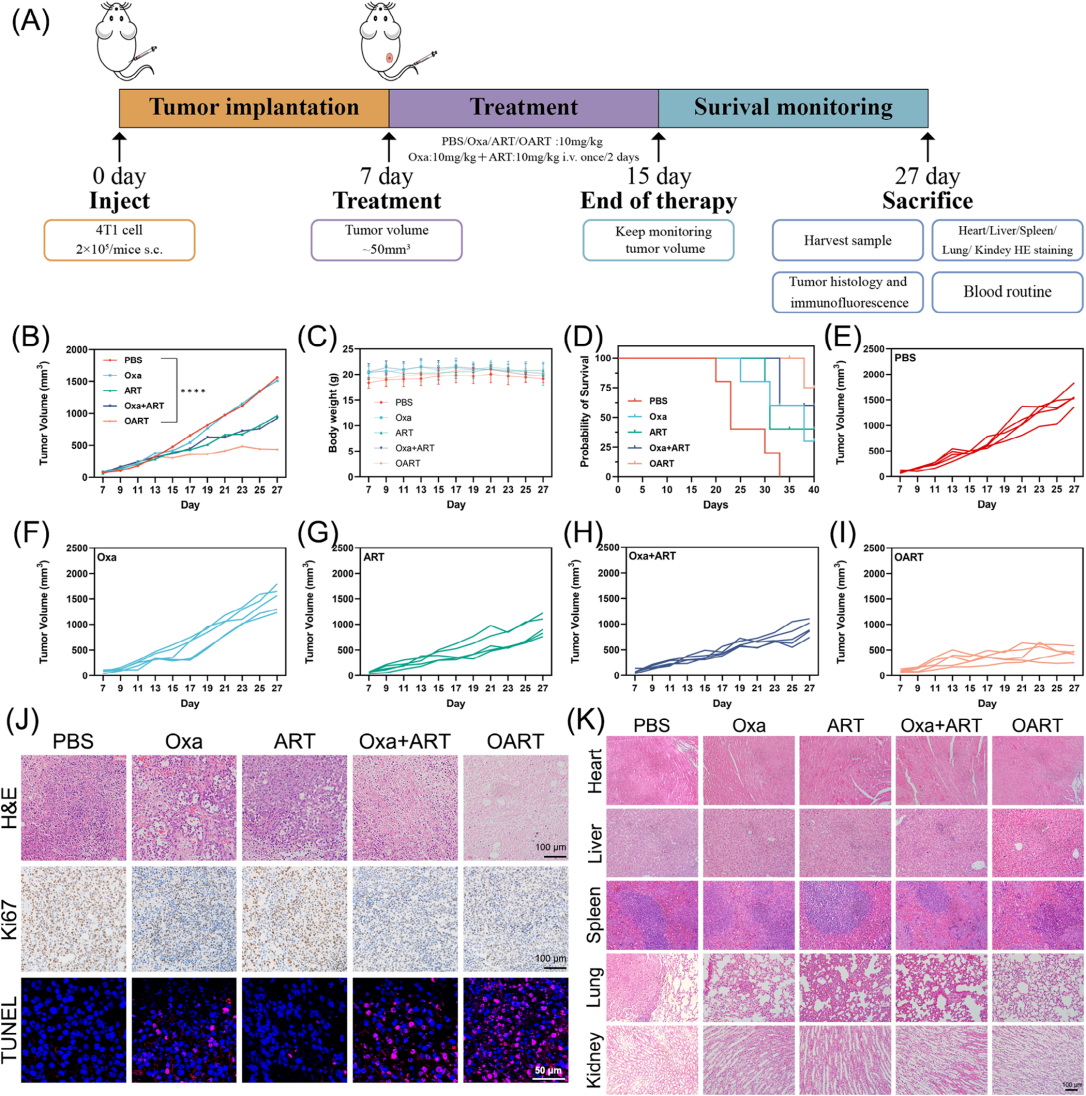
In vivo evaluation of the antitumor efficacy of OART. (A) Administration scheme (*n* = 5). (B) The volume growth curve of the 4T1 tumor. (C) Body weight of mice. (D) Survival of mice in antitumor experiments in vivo (*n* = 5). (E–I) Growth curve of tumors in each group. (J) H&E staining, Ki67, and TUNEL determination on tumor slice. H&E and Ki67 scale bars = 100 μm; TUNEL scale bars = 50 μm. (K) H&E staining for major organs in mice. Data are expressed as mean ± SD. ****p < 0.0001.

To evaluate pharmacological activity in vivo, female Balb/c mice were employed for establishing xenograft model by subcutaneously inoculating 2 × 10^5^ 4T1 cells per mouse in the flank. After tumor volume reached 50 mm^3^, the mice were divided into five groups and administrated the drug as per schedule (Figure 4A). Fifteen days later, the administration was ended up, then we kept monitoring the mice body weight and tumor volume for another 12 days.

As shown in Figure 4B, the OART group exhibited an extraordinary tumor regression effect compared with the PBS group. Furthermore, the therapeutic efficacy of OART (10 mg/kg) treatment was superior to any single dose or combination treatment, while maintaining a stable body weight (Figure 4C). The growth curve of tumors in each group is shown in Figures 4E–I. In addition, the survival curves of the mice during treatment with the different drug can also prove that OART provides a stronger antitumor ability in vivo and has a better prognosis (Figure 4D). The expression of antiferroptosis marker GPX4 in tumor issue demonstrated a decreasing tendency by western blot and immunochemistry staining (Figure S10), confirming the in vivo occurrence of ferroptosis. Hematoxylin and eosin (H&E) staining for tumor slice shows that tumor treated with OART displays fragmented nuclei and shrinkage on nuclei scale, indicating a recession on tumor malignance (Figure 4J). For immunochemistry analysis, we found a decrease on the key proliferation index Ki67 and increasing damaged DNA in tumor, as indicated by red fluorescence signal (Figure 4J).

The safety evaluation on organs after treatment was evaluated by H&E, and no obvious damage was found in liver, heart, spleen, lung, and kidney (Figure 4K). In addition, the analysis of leukocyte subsets, red blood cells, and platelets in blood did not reveal any substantial differences between the OART‐treated group and control group (Figure S11), demonstrating negligible systemic toxicity of OART.

### 2.5 OART amplifies ICD effect and raises immune response

**FIGURE 5:**
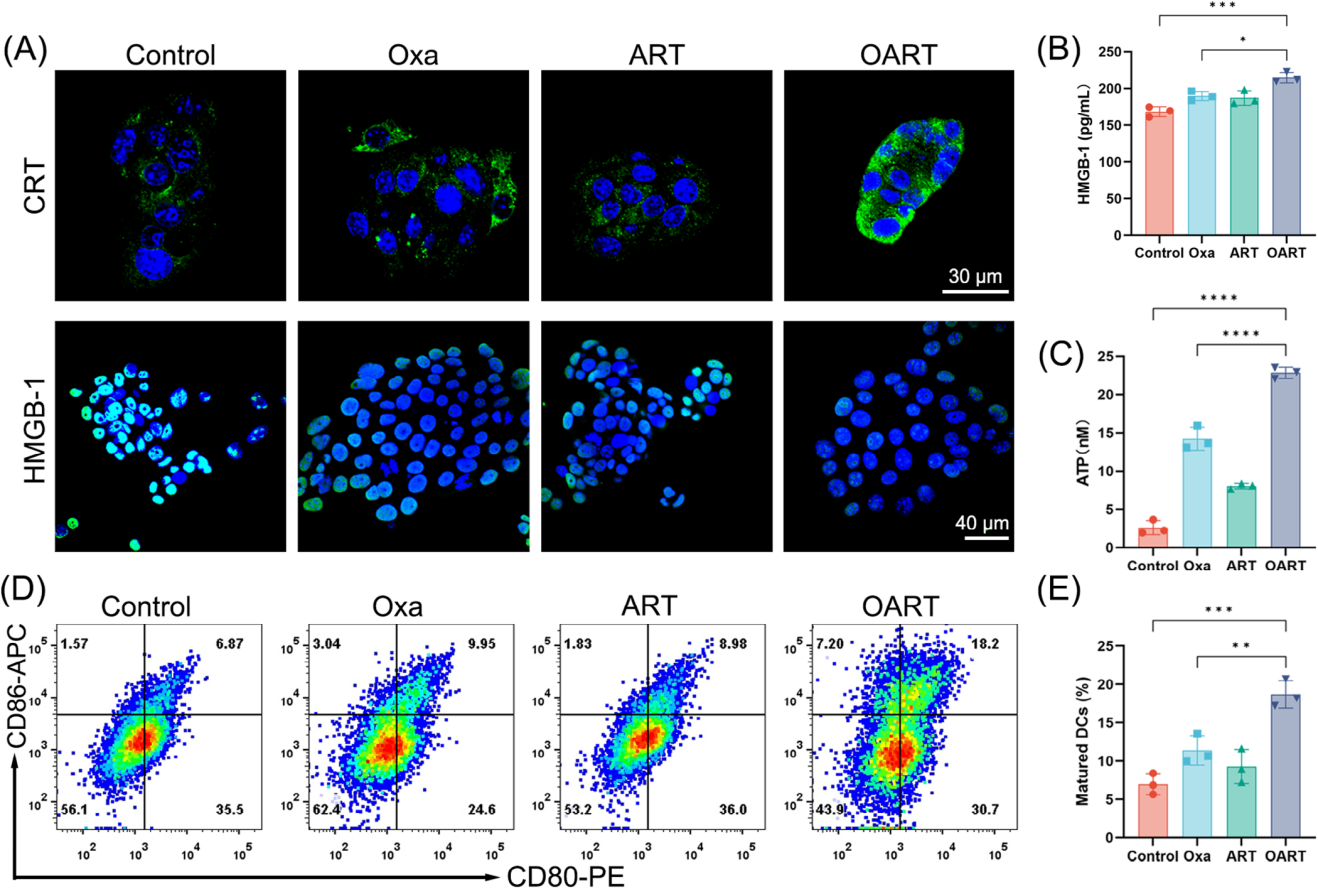
OART raises ICD in vitro. (A) The protein expression of CRT and HMGB‐1. CRT scale bars = 30 μm; HMGB‐1 scale bars = 40 μm. (B) The content of secretory HMGB‐1 captured by ELISA (*n* = 3). (C) Extracellular ATP determined by assay kit (*n* = 3). (D and E) Maturation rate on BMDCs determined by flow cytometry (*n* = 3). Data are expressed as mean ± SD. *p < 0.05, **p < 0.01, ***p < 0.001, ****p < 0.0001.

ICD is the process by which tumor cells transform from nonimmunogenic to immunogenic when they die from external stimuli, such as drugs.^38^ During ICD process, the tumor release signal molecules, known as damage‐associated molecular patterns (DAMPs), including ATP, calreticulin (CRT), and high mobility group box 1 (HMGB‐1). We used CLSM to capture CRT expression, which showed an obvious upregulation and enrichment in membrane (Figure 5A). Meanwhile, an obvious shrinkage on HMGB‐1 expression in nuclei was found in 4T1 cells after treating with OART (Figure 5A). Meanwhile, secretory HMGB‐1 was captured by ELISA, and we observed a slight increase (Figure 5B). Extracellular adenosine triphosphoric acid (ATP) leakage sends a “find me” signal that promotes the recruitment of antigen presentation cells.^6^, ^25^ The ATP luminescence assay reveled that extracellular ATP concentration dramatically increased after treatment especially in OART group (Figure 5C).

As a classical category antigen‐presenting cells, dendritic cells derived from bone marrow (BMDCs) were cultured to evaluate the immune activation in vitro. The extracellular medium from 4T1 cells treated with the indicated drugs was collected to stimulate BMDCs for 24 h, and its maturation was detected by flow cytometry (Figure 5D). The percentage of mature DCs (CD80^+^, CD86^+^) increased from 6.87% to 18.2%, as shown in in Figure 5E, which was much higher than that of any single dose. Overall, these results confirm the occurrence of ICD caused by ferroptosis in vitro.

**FIGURE 6:**
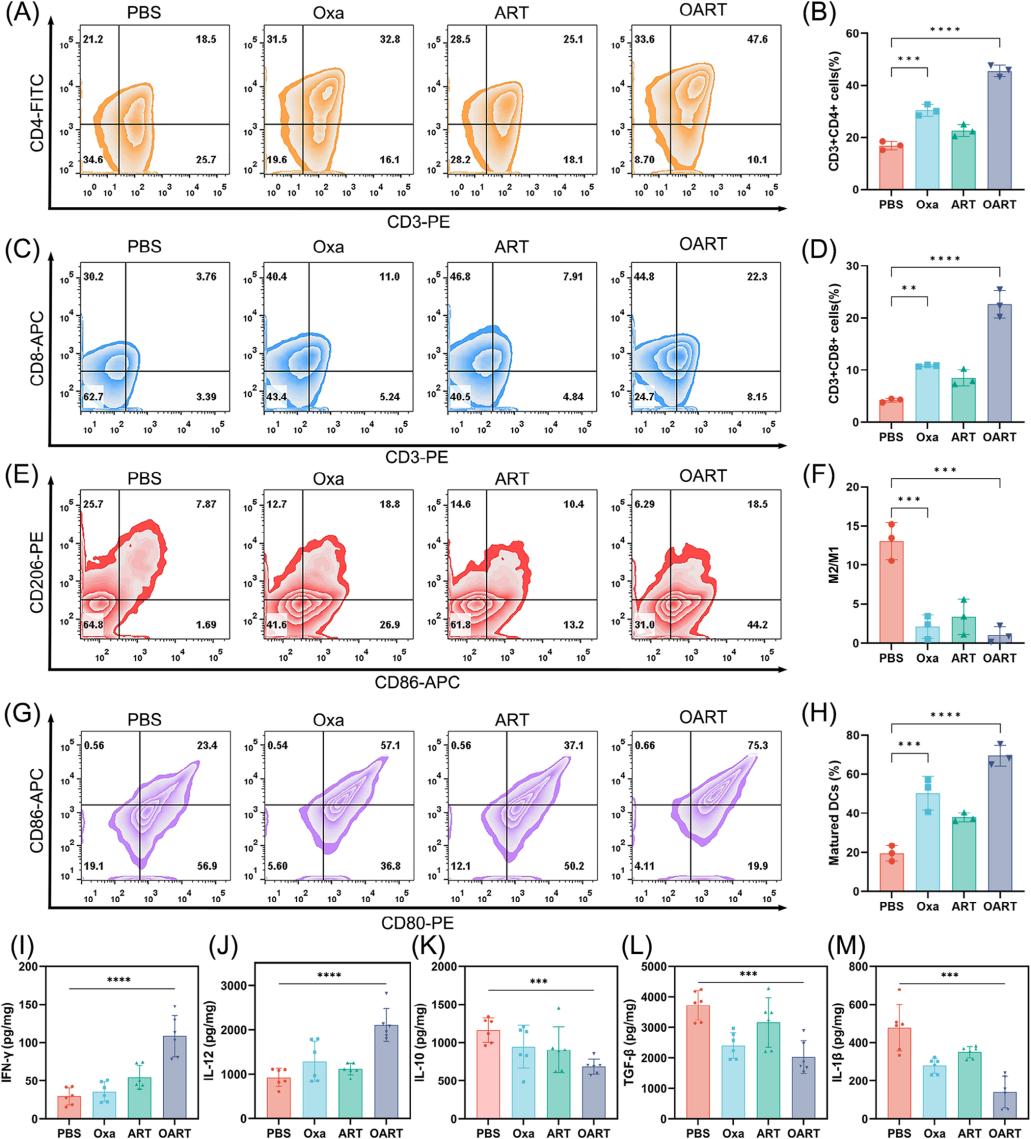
OART amplifies ICD effect and raises immune response in vivo. (A and B) Determination on activation of T helper (CD3+ CD4+) cells in tumor tissue by flow cytometry (*n* = 3). (C and D) Determination on activation of cytotoxic T cells (CD3+ CD8+) in tumor tissue by flow cytometry (*n* = 3). (E and F) Determination on phenotype of tumor associated macrophages (M1 marker: CD86, M2 marker: CD206) in tumor tissue by flow cytometry (*n* = 3). (G and H) Characterization on maturation of DCs (CD80+ CD86+) tumor tissue by flow cytometry (*n* = 3). (I–M) Cytokine secretion in tumor tissue (*n* = 6). Data are expressed as mean ± SD. **p < 0.01, ***p < 0.001, ****p < 0.0001.

For in vivo immune response, we employed flow cytometry to determine the components of immunocytes subsets in tumor tissue. As is shown in Figures 6A and B, CD3^+^ CD4^+^ T helper lymphocyte increased from 18.5 to 47.6% and the percentage of cytotoxic T lymphocytes marked with CD3^+^ CD8^+^ elevate from 3.76 to 22.3% (Figures 6C and D). These results suggest that OART activates the T lymphocyte cells in vivo. Tumor‐associated macrophages (TAMs) are important innate immune cells that differentiate into two phenotypes with distinct functions: an M1‐like phenotype and an M2‐like phenotype. M1‐like TAMs exert antitumor effects by engulfing tumor lysis, whereas TAMs in M2‐like phenotype exhibit tumorigenic activity which is actively involved in tumor proliferation and metastasis. Therefore, we use flow cytometry to determine the phenotype of TAMs in tumors. Figures 6E and F show that the percentage of M2/M1 dramatically decreased, after treatment with OART, indicating a reversal of the phenotype. To evaluate antigen presentation, we determined the DCs content of the tumors. The percentage of mature DCs, characterized by dual‐positive CD86 and CD80 signals, increased from 23.4 to 75.3% in the CD11c^+^ cell subset of the tumor (Figures 6G and H).

Besides, cytokines were determined to evaluate the function of immunocytes in the tumor microenvironment. The increase on interferon‐γ (IFN‐γ) content suggests an enhancement on T lymphocyte function (Figure 6I). Interleukin‐12 (IL‐12) is a key promoter of IFN‐γ,^39^ and its concentration was found elevated in tumor, indicating a proinflammatory signature (Figure 6J). IL‐10 and transforming growth factor‐β (TGF‐β) were found downregulated in OART group, consistent with decreased M2 phenotype TAMs, indicating an immune reverse in tumor microenvironment (Figures 6K and L). Also, IL‐1β was also determined and the result showed that OART was able to shrink the expression tumorigenic cytokine (Figure 6M).

In conclusion, we found that OART increases tumor immunogenicity by triggering ICD, improves the immune response by promoting T lymphocyte maturation, reversing M2‐like macrophages, and enhances tumor‐killing cytokine secretion in tumors.

### 2.6 OART inhibits lung metastasis and improve response of αPD‐L1 in vivo

The main challenges in clinical treatment in triple negative breast cancer are tumor metastasis and low immune response.^40^ Since 4T1 cells are derived from triple‐negative breast cancer and are characterized as high metastatic and low immunogenic types,^41^ we established an orthotopic breast cancer model accompanied with lung metastasis to mimic the clinical scenario. Given that OART had strong immunogenic effects on 4T1 cells, we aimed to investigate whether the immune effect activated by OART could prevent lung metastasis.

**FIGURE 7:**
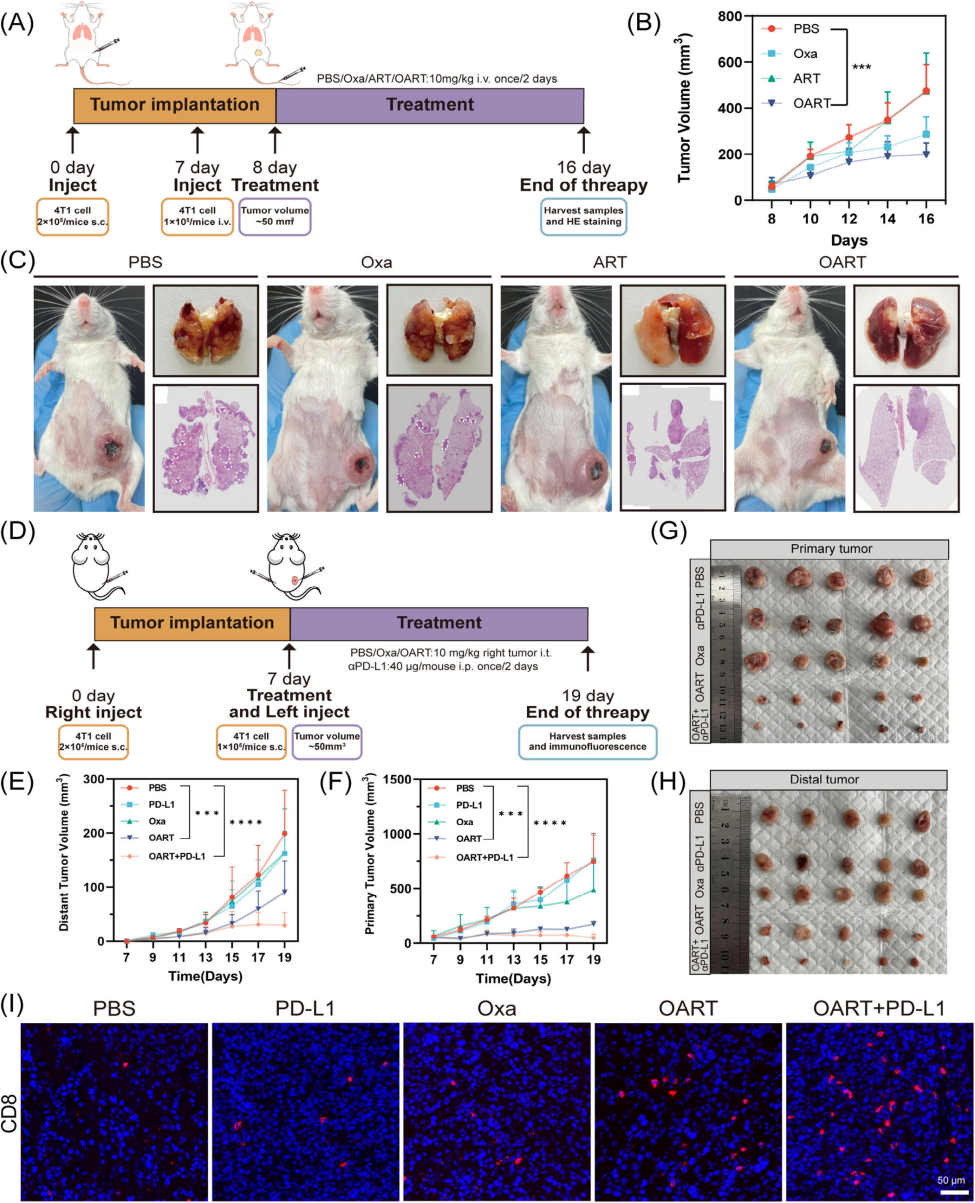
OART inhibits lung metastasis and improves the response of αPD‐L1 in vivo. (A) Administration schedule (*n* = 5). (B) Growth curve on orthotopic tumors of mice during therapy. (C) OART inhibit tumor growth in orthotopic breast cancer model accompanied with lung metastasis. (D) Administration schedule on PD‐L1 combination therapy (*n* = 5). (E–H) Growth curves and images on primary distal tumors. (I) CD8+ T lymphocytes infiltration in distal tumors. Scale bars = 50 μm. Data are expressed as mean ± SD. ***p < 0.001, ****p < 0.0001.

To evaluate antitumor metastasis in vivo, we inoculated seeded 2 × 10^5^ 4T1 cells in the mammary fat pad to imitate a breast cancer in situ model, then, 1 × 10^5^ 4T1 cells via tail vein injection to simulate the metastasis of breast cancer. Following the administration scheme (Figure 7A), the mice were treated with 10 mg/kg Oxa, ART, or OART once every other day. We then monitored the tumor volume in situ and recorded mouse body weight. As shown in Figure 7B, OART exerted a potent regression on orthotopic tumor growth, while tumors in the PBS group grew rapidly. Single administration of Oxa or ART mildly delayed tumor regression but showed lower therapeutic efficacy than OART. The body weight of the mice showed no significant difference among the four groups (Figure S12A). Two weeks later, we harvested lung samples when mice were under dyspnea. The image of pulmonary nodules was photographed and the section of lungs were analyzed by H&E staining. As Figures 7C and S12C depicted, there were large number of aggressive lung foci formed in the PBS group, while the nodules scaled back in administrating group. Treating with OART extremely diminished the metastasis foci. H&E staining further demonstrated that OART reduced nodule number and inhibited tumor metastasis. In addition, as shown in Figure S12B, the survival of mice was significantly increased after OART treatment. Above all, OART showed strong potential on tumor metastasis.

Low immunogenicity tends to cause an insufficient response to immune checkpoint blockade, posing a challenge for breast cancer immunotherapy.^40^ Considering that OART elevated tumor immunogenicity, we explored the therapeutic efficacy of combination therapy with αPD‐L1 by establishing a bi‐lateral tumor model in mice. As illustrated in Figure 7D, each mouse was inoculated with 2 × 10^5^ 4T1 cells into the right flank. After 7 days, 1 × 10^5^ 4T1 cells were subcutaneously inoculated into the left flank to simulate the distal metastases. We set five groups for administration: PBS, Oxa, αPD‐L1, OART, combination on OART and αPD‐L1. We observed a noticeable tumor recession in both the primary tumor and distal tumor in the administration group (Figures 7E–H). Tumor growth curves revealed that the combination group displayed the strongest tumor suppression compared with the PBS group (Figures S13 and S14). Intratumor administration of Oxa alone inhibited primary tumor growth, but had limited effect on the distal tumor. A single administration of αPD‐L1 exerted a moderate effect on distal tumors but had no influence on the primary tumor, indicating an effective but limited systemic influence on tumor. Combination administration exhibited more powerful therapeutic efficacy, even though OART monotherapy had a comparable potent antitumor effect. Inspired by the successful outcome on the combination therapy trial, we conducted further evaluation on the infiltration of T lymphocyte cells into distal tumors. The subpopulation of tumor infiltrating T cells was analyzed by immunofluorescence (IF) staining. As Figure 7I demonstrated, the percentage of CD8^+^ cells in distal tumors was dramatically increased in combination group, which solidified its effect on immune modulation after administration.

## 3 DISCUSSION

Immunotherapy is an emerging antitumor strategy, but the core problem for its antitumor activity lies in the deficient immunogenicity of tumor cells.^42^ Poor immune response tends to increase the risk of tumor metastasis and relapse. Ferroptosis was once deemed as a candidate to solve this problem. Tumor cells dying from ferroptosis release DAMPs to elevate immunogenicity and contributes to ICD response. As a powerful chemotherapeutic agent that has been widely used in clinical cancer therapy, Oxa depends on ICD to exert immune modulations.^43^ However, the sustainability of ICD is limited by the high GSH concentration in tumor environment.^44^ Hence, utilizing ferroptosis to deplete GSH is able to compensate for the disadvantage of Oxa and amplify ICD in tumor.

This study showed that the complementary combination of Oxa and ART finally improves drug efficiency and significantly enhances immune response in tumor. Single ART treatment indeed exhibits a tumor‐repressed effect in mice, but the immune response in vitro and vivo was less active. By introducing Oxa, OART significantly inhibited lung metastasis and abscopal tumors and improved the efficacy of αPD‐L1 therapy.

Regarding the mechanism of action, mitochondria were found to be the main location for LPO in the process of ferroptosis, except for the cell membrane. Specifically, OART downregulates mitochondrial fusion and fission, thereby producing massive ROS and amplifying LPO. In addition, DHODH serves as a ferroptosis defender in mitochondria by producing panthenol to defend against LPO instead of GSH. Consequently, downregulating DHODH by OART disrupts redox balance in the mitochondria and makes tumor cells more vulnerable to ferroptosis. Transcriptome analysis revealed that OART influence genes related with mitochondrial membrane, nucleoid, translocation, and so on. These findings are highly consistent with previous research that ART interacts with mitochondria‐related proteins and regulates mitochondrial dynamics.^45^, ^46^ Evaluating proteins related to mitochondrial dynamics demonstrates that OART destroys membrane integrity and regulates mitochondrial fusion and fission. OART is the first small‐molecule ferroptosis inducer to establish linkage between mitochondrial dynamics and redox balance during the ferroptosis. Such unprecedented findings replenish current mechanism on ferroptosis and it would be meaningful to carried further exploration.

The major restriction of applying ferroptosis in tumor therapy lies in immune suppression caused by excessive peroxide leakage. In this study, OART is designed from the clinically used chemotherapeutics Oxa and antimalarial drug ART. Both of them have no safe no concerns, which makes OART entitles with clinical suitability. The platinum(IV) strategy has been developed to compensate for the intrinsic disadvantage of traditional platinum therapy by introducing functional ligands.^47^, ^48^, ^49^ Over the past few decades, multiple Pt(IV) complex have been exploited to overcome drug resistance, improve drug lipophilicity, and prolong metabolic half‐time.^50^ There is no doubt Pt(IV) strategy is a feasible and promising therapeutic schedule. Among previous reports in Pt(IV) complex, most researchers focus on how to kill tumors, but neglect immune modulation in the tumor microenvironment. ART is a classical antimalarial drug that has successfully unleashed numerous patients who suffer from malaria.^51^, ^52^ As a great medicine in 21st century, ART also downregulates the core homologous recombination protein RAD51,^42^ thereby improving sensibility to DNA damage agents. This offers a chance to develop novel Pt(IV) complex. Different from utilizing ART to address drug resistance problem, we concentrate on its powerful capacity on inducing ferroptosis and contribution to improve tumor immunogenicity. Recently, a report reveals a novel mechanism that ART inhibits the activity of TREM2 in macrophages and suppresses on tumor growth.^53^ Though diverse and independent survey we carried, we are the same end, which solidifies that OART is a feasible immunomodulatory agent.

## 4 CONCLUSION

**FIGURE 8:**
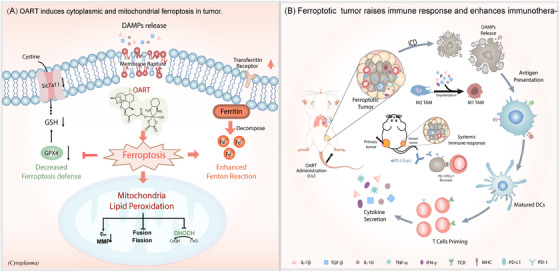
Schematic illustration on mechanism. (A) OART induces cytoplasmic and mitochondrial ferroptosis in tumor and release DMAPs. (B) Ferroptotic tumor raises immune response and enhance immunotherapy.

In this paper, we have developed a ferroptosis‐inducer OART with a substantial ferroptotic bioactivity in vitro and vivo with novel mechanism. As Figure 8 depicts, OART induces cytoplasmic and mitochondrial LPO to promote tumor ferroptosis. For details, OART inhibits ferroptosis defense system by depleting GSH and downregulating the protein levels of GPX4 as well as SLC7A11. Meanwhile, OART enhances Fenton reaction by decomposing ferritin, upregulating TFR, and elevating intracellular iron concentration. Strikingly, OART induces mitochondrial LPO by inhibiting mitochondrial fission and fusion, destroying MMP, and downregulating DHODH. The redox imbalance causes massive ROS leakage, thereby catalyzing a continuous LPO process. The ferroptotic cells release more DAMPs to enhance immune response by promoting DCs maturation, T cells priming, and immune‐cytokines secretion as well as TAMs reprogramming. In vivo, OART exerts strong tumor regression in tumor‐bearing mice.

To further evaluate its potential for clinical application, multiple tumor cancer models were established to determine the antitumor effects of OART therapy. We employed a primary breast cancer model in mice, accompanying with lung metastasis, and found a decrease in tumor growth and pulmonary foci, indicating the potential of OART on inhibiting tumor metastasis. In a bilateral tumor model, intratumoral injection of OART in primary tumor elicits a systemic immune response and inhibits distal tumor development. Synergistic therapy with αPD‐L1 demonstrates additive effect on tumor regression, confirming its potential on clinical translation.

## 5 MATERIALS AND METHODS

### 5.1 Chemical agents and antibodies

All commercial reagents were used without purification unless otherwise specified. Oxa and ART were purchased from Sigma–Aldrich and used as received without further purification. O‐(benzotriazol‐1‐yl)‐N,N,Nʹ,Nʹ‐tetramethyluronium tetrafluoroborate, triethylamine, N‐dimethylformamide, dimethyl sulfoxide (DMSO) and hydrogen peroxide (30%) were purchased from J&K Scientific. Flash‐column chromatography was realized with 200−300 mesh silica gel (Qingdao Haiyang Chemical, China).

MTT, Calcein/PI Cell Viability/Cytotoxicity Assay Kit, Crystal Violet Staining Solution, 4% paraformaldehyde, RIPA lysis buffer, PMSF, phosphatase inhibitor cocktail A, enhanced BCA Protein Assay Kit, enhanced MMP assay kit with JC‐1, BeyoECL Star, Lipid Peroxidation MDA Assay Kit, GSH and GSSG Assay Kit, and enhanced ATP Assay Kit were purchased from Beyotime, Shanghai. NucBlue™ Fixed Cell ReadyProbes™ (DAPI), MitoTracker™ Orange, ActinGreen™ 488 ReadyProbes™, and C11‐BODIPY^581/591^ were purchased from Thermo Scientific. Tom20, OPA1, MFF, anti‐mouse IgG, and HRP‐linked antibody were purchased from Cell Signaling Technology, Inc. GAPDH, GPX4, TFR, SLC7A11, Ferritin HC, ALOX5, and Goat Anti‐Rabbit IgG H&L (HRP) were purchased from Abcam. DHODH was purchased from Proteintech. Cy3‐conjugated goat anti‐mouse IgG (H+L) was purchased from Servicebio. Granulocyte‐macrophage colony‐stimulating factor (GM‐CSF) and IL‐4 were bought from Sangon Biotech, Shanghai. Recombinant cytokine lysis diluent set were purchased from Multisciences, Hangzhou. HMGB‐1, IL‐10, IL‐12, IL‐1β, TGF‐β, and IFN‐γ were purchased from Shanghai, Fanke Industrial Co., Ltd. InVivoMab anti‐mouse PD‐1 (CD279) was purchased from BioXcell.

### 5.2 Cell culture

The murine breast cancer 4T1 (Cell Bank of the Chinese Academy of Sciences, China) was cultured in the complete RPMI‐1640 medium (Sangon Biotech; E600028) including 10% fetal bovine serum (FBS; Excell Bio, Shanghai; FSP500) and 1% penicillin–streptomycin 100× (PS; Macgene, Beijing, CC004). The murine melanoma B16F10 (Cell Bank of the Chinese Academy of Sciences, China) was cultured in the complete DMEM medium (high glucose; Sangon Biotech; E600003) including 10% FBS and 1% penicillin–streptomycin 100×. The murine kidney cancer Renca was purchased from Procell (Wuhan; CL‐0568) and incubated with complete RPMI‐1640 (Procell, Wuhan; PM150110) with 10%FBS, 1% penicillin–streptomycin 100x, 1% MEM Nonessential Amino Acids Solution 100× (NEAA; Macgene; CC25025), 1 mM Sodium Pyruvate (Macgene; CC007), and 2 mM L‐glutamine (Macgene; CC009). The human normal endothelial cell line PUMC‐HUVEC‐T1 was cultured in the DMEM medium (high glucose) and added 1%NEAA and insulin (Macgene).

All the cells were incubated in their special medium and maintained in a carbon dioxide cell culture box (Thermo Fisher‐Forma 371) with 5% CO_2_ at 37°C.

### 5.3 | In vitro cytotoxicity assays

The stock solutions of platinum(IV) complex OART and prototype drugs Oxa and ART were prepared in DMSO. Cytotoxicity was tested on murine breast cancer (4T1), murine melanoma (B16F10), and murine kidney cancer (Renca) cell lines via MTT assay. The toxicity of the compounds to normal cells was detected on the human normal endothelial cell line PUMC‐HUVEC‐T1. All kinds of cells were seeded in a 96‐well plate (Guangzhou Jet Bio‐filtration Co., Ltd.) with a density of 5 × 10^3^ cells per well in a 200 μL growth medium and preincubated for 12 h then removed medium and replaced complex solution (200 μL per well, DMSO < 0.1%). After exposure of 48 h, 20 μL MTT solution (5 mg/mL in ddH_2_O, ST316; Beyotime Biotechnology) was added to the well and incubated for 4–6 h at 37°C. After that, the culture medium was removed and added in 200 μL DMSO to dissolve the purple MTT‐formazan crystals. The plates were shaken for 20 min at room temperature and the absorbance of the solution at 490 nm was measured on a microporous plate multifunctional detector (Tecan‐Tecan Spark, Switzerland).

### 5.4 Western blot

For western blot assay, cells were seeded in a six‐well plate and incubated with Oxa, ART, and OART (2 μM) for 24 h. After the end of the experiment in vivo, the tumor tissue was collected and stored at −80°C. Then, all the samples both cell and tumor tissue were extracted and homogenized by RIPA lysis buffer. The lysates were centrifuged at 13000 g for 10 min at 4°C. Protein extracts were separated by sodium dodecyl sulfate‐polyacrylamide gel electrophoresis and transferred to 45 μm polyvinylidene difluoride (Millipore) membranes. Blocked with 5% nonfat milk (P0216; Beyotime) in TBS‐0.1%Tween 20 (TBST) for 2 h at room temperature. The membranes were then incubated with diluent primary antibodies overnight at 4°C. Antibodies and working diluted concentrations were GAPDH (Abcam; ab215191, 1:10,000), DHODH (Proteintech; 14877‐1‐AP, 1:1000), Tom20 (CST; 42406, 1:1000), OPA1 (CST; 80471, 1:1000), MFF (CST; 84580, 1:1000), and GPX4 (Abcam; ab125066, 1:5000).

### 5.5 Intracellular ROS generation measure

Intracellular ROS generation was measured by flow cytometry and CLSM. 4T1 cells were seeded in a six‐well or 12‐well plate and incubated with Oxa, ART, and OART (2 μM) for 24 h. ROS generation was detected by a fluorescent dye DCFH‐DA. Before cell stain, cells were washed two times with PBS, using 2 μM DCFH‐DA solution incubated for 30 min at 37°C. Then, used the flow cytometry and confocal microscope to detect the mean fluorescence intensity

### 5.6 LPO assay

Cellular LPO level was evaluated by C11‐BODIPY^581/591^ dye (ThermoFisher Scientific; M7510) and lipid peroxidation MDA assay kit (Beyotime; S0131S). Cells were seeded in a six‐well or 12‐well plate and incubated with Oxa, ART, and OART (2 μM) for 24 h. Then, trypsinized the cell from the six‐well plate and assessed MDA concentration by lipid peroxidation MDA assay kit following the manufacturer's procedure. Then, the absorbance of the solution at 532 nm was measured on a microporous plate multifunctional detector. For the C11‐BODIPY^581/591^ staining assay, cells were washed with cold PBS after incubated drug solutions and changed from the cultured medium to the fresh medium containing C11‐BODIPY^581/591^ dye (2 μM) for 30 min at 37°C. Finally, used the confocal microscope to image mitochondria activation.

### 5.7 RNA‐seq

Total RNA of 4T1 cells from four different experiments treated with DMSO (control), Oxa, ART, and OART (2 μM) for 24 h was collected by Trizol (ThermoFisher; 15596018) following the manufacturer's procedure and purified and measured by Bioanalyzer 2100 and RNA 6000 Nano LabChip Kit (Agilent, CA, USA; 5067‐1511) high‐quality RNA samples with RIN number >7.0 were used to construct sequencing library.

RNA‐seq was performed by LC Sciences through the Illumina X10 platform (Hangzhou, Zhejiang, China). Genes with The genes with the parameter of false discovery rate below 0.05 and absolute fold change≥2 were considered differentially expressed genes. Differentially expressed genes were then subjected to enrichment analysis of GO functions and KEGG pathways. The correlation among all samples was detected by Pearson correlation analysis and PCA. Volcano analysis was used to identify the DEGs between the treated and control groups.

### 5.8 Immunofluorescence

IF staining on cell and tumor tissue sections. Seeded the 4T1 tumor cells into confocal dishes or growing on glass coverslips in the 12‐well plate. Oxa, ART, and OART (2 μM) were added and incubated for 24 h. The tumor tissue was sliced by paraffin embedding technique. Cell and tissue samples were fixed in 4% paraformaldehyde for 10 min and permeabilized in 0.1% Triton X‐100 solution for 10 min (optional). Incubated the primary antibodies with the appropriate concentration, 4°C overnight. Then, incubated the secondary antibodies conjugated with different fluoresceins at room temperature for 1 h. Finally, samples were incubated with DAPI and analyzed by confocal microscopy. The antibodies and their brand used in IF experiment: anti‐CRT (Abcam; ab92516, 1:500), anti‐HMGB‐1 (Abcam; ab18256, 1:1000), and goat anti‐rabbit IgG H&L (Alexa Fluor® 488) (Abcam; ab150077, 1:1000)

### 5.9 Maturation of BMDCs

BMDCs were extracted from 6‐week‐old female C57BL/6 mice and cultured in complete RPMI 1640 medium supplement with GM‐CSF (20 ng/mL; Sangon Biotech) and IL‐4 (10 ng/mL; Sangon Biotech) at 37°C with 5% (v/v) CO_2_. After incultured for 7−10 days, cells could conduct subsequent experiments.

4T1 cells and BMDCs were respectively seeded in a six‐well plate. 4T1 cells were treated with different drugs for 24 h, then BMDCs were cultured with supernatant of 4T1 cells for 24 h. Subsequently, the dendritic cells were stained with the corresponding antibody (anti‐CD11c‐FITC, anti‐CD80‐PE, and anti‐CD86‐APC) for 1 h at 4°C. The maturation of the dendritic cells was detected by flow cytometry.

### 5.10 Antitumor efficacy study and biosafety evaluation

Six‐week‐old healthy BALB/C and C57Bl/6 female mice (weight, 18–20 g) were purchased from Charles River, Beijing. All the animals were housed in a pathogen‐free condition in groups of 5−6 mice per cage, and the temperature was kept at 21  ±  2°C, and the relative humidity was maintained at 40−70% with a 12 h light/dark cycle. Before the experiment, fed these mice in the stable environment for at least 1 week. All the procedures followed the guidelines of the Institutional Animal Care and Use Committee at Northwestern Polytechnical University.

Anti‐breast cancer research in vivo was studied in BALB/C mice, 2 × 10^5^ 4T1 tumor cells were subcutaneously inoculated in the BALB/C mice, when the primary tumors reach the size of 50 mm^3^, randomly divided these mice into five groups and each group contained five mice: PBS, Oxa, ART, Oxa+ART, and OART (i.v. 10 mg/kg, once every 2 days). The body weight and tumor volume of the mice were continuously monitored during the experiment., The mice were sacrificed on the 27th day, collected the main tissues (heart, liver, spleen, lung, and kidney) for H&E staining, and the blood was analyzed by blood routine to evaluate the biosafety of the drug. Finally, the tumors were also harvested for immunohistochemistry (Ki‐67), TUNEL, and H&E staining.

### 5.11 In vivo immune activation

To determine the ability of OART to activate the immune system in vivo, we conducted experiments on BALB/C mice. First, the orthotopic tumor model of breast cancer was constructed on the mice and three performed three times of various drug therapy. Then separated the tumor tissue of the mice and produced a single‐cell suspension. Finally, stained cell with antibody and performed flow cytometry. The antibodies used in the experiment were as follows: anti‐CD3‐PE, anti‐CD4‐FITC, anti‐CD8‐APC, anti‐CD45‐Percp‐Cy5.5, anti‐CD11c‐FITC, anti‐CD80‐PE, anti‐CD86‐APC, and anti‐CD206‐PE.

### 5.12 Inhibition of lung metastasis and synergistic PD‐L1

Breast cancer lung metastasis model was studied in BALB/C mice, 2 × 10^5^ 4T1 tumor cells were subcutaneously inoculated near the mammary gland of the BALB/C mice, when the primary tumors reach the size of 50 mm^3^, randomly divided these mice into four groups and each group contained five mice: PBS (i.v. 10 mg/kg, once every 2 days), Oxa (i.v. 10 mg/kg, once every 2 days), ART (i.v. 10 mg/kg, once every 2 days), and OART (i.v. 10 mg/kg, once every 2 days). Before the treatment, 1 × 10^5^ 4T1 cells were injected intravenously to make a lung metastasis model. The mice were routinely monitored for tumor volumes and body weight. The mice were sacrificed on day 16 for lung H&E staining assays.

When the tumors reached approximately 50 mm^3^ were divided randomly into five groups (*n* = 5 per group) for administration: PBS (i.t. 10 mg/kg, once every 2 days), Oxa (i.t. 10 mg/kg, once every 2 days), αPD‐L1(i.p. 40 μg/mouse, once every 3 days), OART (i.t. 10 mg/kg, once every 2 days), combination on OART and αPD‐L1 (i.t. 10 mg/kg, once every 2 days and i.p. 40 μg/mouse, once every 3 days). The tumor volumes and body weight of each mouse were recorded every 2 days. After the treatment, tumor tissues in distal were sliced for IF staining.

### 5.13 Statistical analysis

Data were expressed as means ± SD. For comparison between groups, statistical differences were analyzed two‐sided unpaired t tests. For multiple sample analyses, statistical differences were analyzed by one‐way ANOVA with Tukey's multiple comparisons. All statistical analyses were performed using GraphPad Prism version 9 software. Statistical significance was defined as *p* < 0.05. Levels of significance were indicated as *p < 0.05, **p < 0.01, ***p < 0.001.

## AUTHOR CONTRIBUTIONS

*Conceptualization*: Gaofei Wei, Le Yang, and Minggao Zhao. *Methodology*: Renming Fan, Aohua Deng, Ruizhuo Lin, Shuo Zhang, Caiyan Cheng, Junyan Zhuang, and Yongrui Hai. *Writing—origin draft*: Aohua Deng and Renming Fan. *Writing—review and editing*: all authors. All authors have read and approved the final paper.

## CONFLICT OF INTEREST STATEMENT

The authors declare no conflict of interest.

## ETHICS STATEMENT

All the procedures followed the guidelines of the Institutional Animal Care and Use Committee at Northwestern Polytechnical University (Approve number: NPU202101057)

## Supporting information

Supporting Information

## Data Availability

All data and materials are available from the corresponding authors upon request once published.
