# Supplementary material for: A platinum(IV)–artesunate complex triggers ferroptosis by boosting cytoplasmic and mitochondrial lipid peroxidation to enhance tumor immunotherapy
Source: MedComm (2020). 2024 May 20;5(6):e570. doi: 10.1002/mco2.570 (PMC11106517; doi:10.1002/mco2.570)
Supplement: Supplementary file 1 — Supporting Information [file MCO2-5-e570-s001.docx]

Supplementary information

**A platinum(IV)-Artesunate complex triggers ferroptosis by boosting cytoplasmic and mitochondrial lipid peroxidation to enhance tumor immunotherapy**

Renming Fan^1, 2^, Aohua Deng^1, 2^, Ruizhuo Lin^1, 2^, Shuo Zhang^1, 2^, Caiyan Cheng^1, 3^, Junyan Zhuang^1, 2^, Yongrui Hai^1, 2^, Minggao Zhao^3^, Le Yang^3*^, Gaofei Wei^1, 2*^

1. Institute of Medical Research, Northwestern Polytechnical University, Xi’an 710072, China.
2. Research & Development Institute of Northwestern Polytechnical University in Shenzhen, Shenzhen 518057, China.
3. Precision Pharmacy & Drug Development Center, Department of Pharmacy, Tangdu Hospital, Air Force Military Medical University, Xi’an 710038, China.

Renming Fan, Aohua Deng, and Ruizhuo Lin have equal contribution to this study.

**Table of content**

[Figure S1. ^1^H spectrum of OART. 3](#_Toc154605280)

[Figure S2. ^13^C spectrum of OART. 3](#_Toc154605281)

[Figure S3. HRMS of OART. 4](#_Toc154605282)

[Figure S4. HPLC of OART. 4](#_Toc154605283)

[Figure S5. Time dependent morphological observation. 5](#_Toc154605284)

[Figure S6. OART inhibit cell migration in 24 h. 5](#_Toc154605285)

[Figure S7. OART's ability to induce apoptosis and DNA damage. 6](#_Toc154605286)

[Figure S8. Ferroptosis inhibitor rescue experiment. 6](#_Toc154605287)

[Figure S9. OART treatment causes multiple genes changed. 7](#_Toc154605288)

[Figure S10. OART downregulates GPX4 expression in tumor tissue. 7](#_Toc154605289)

[Figure S11. Blood component analysis in mice treated with indicated drugs. 8](#_Toc154605290)

[Figure S12. Body weight changes and survival in mice with lung metastases. 8](#_Toc154605291)

[Figure S13. Image of bilateral tumors in mice with indicated drugs. 9](#_Toc154605292)

[Figure S14. Growth curves of Image of bilateral tumors in mice with indicated drugs. 9](#_Toc154605293)


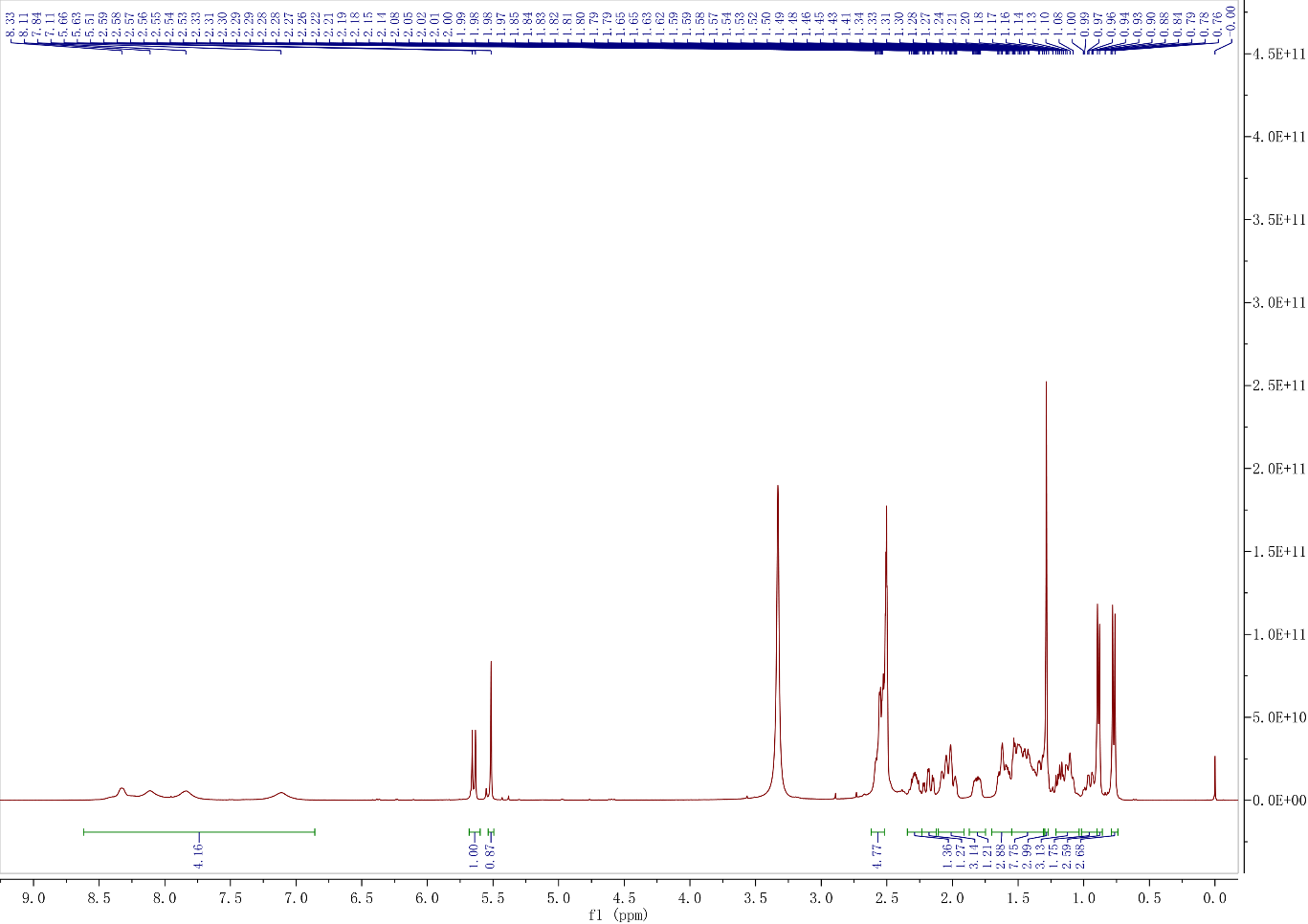


# Figure S1. ^1^H spectrum of OART.


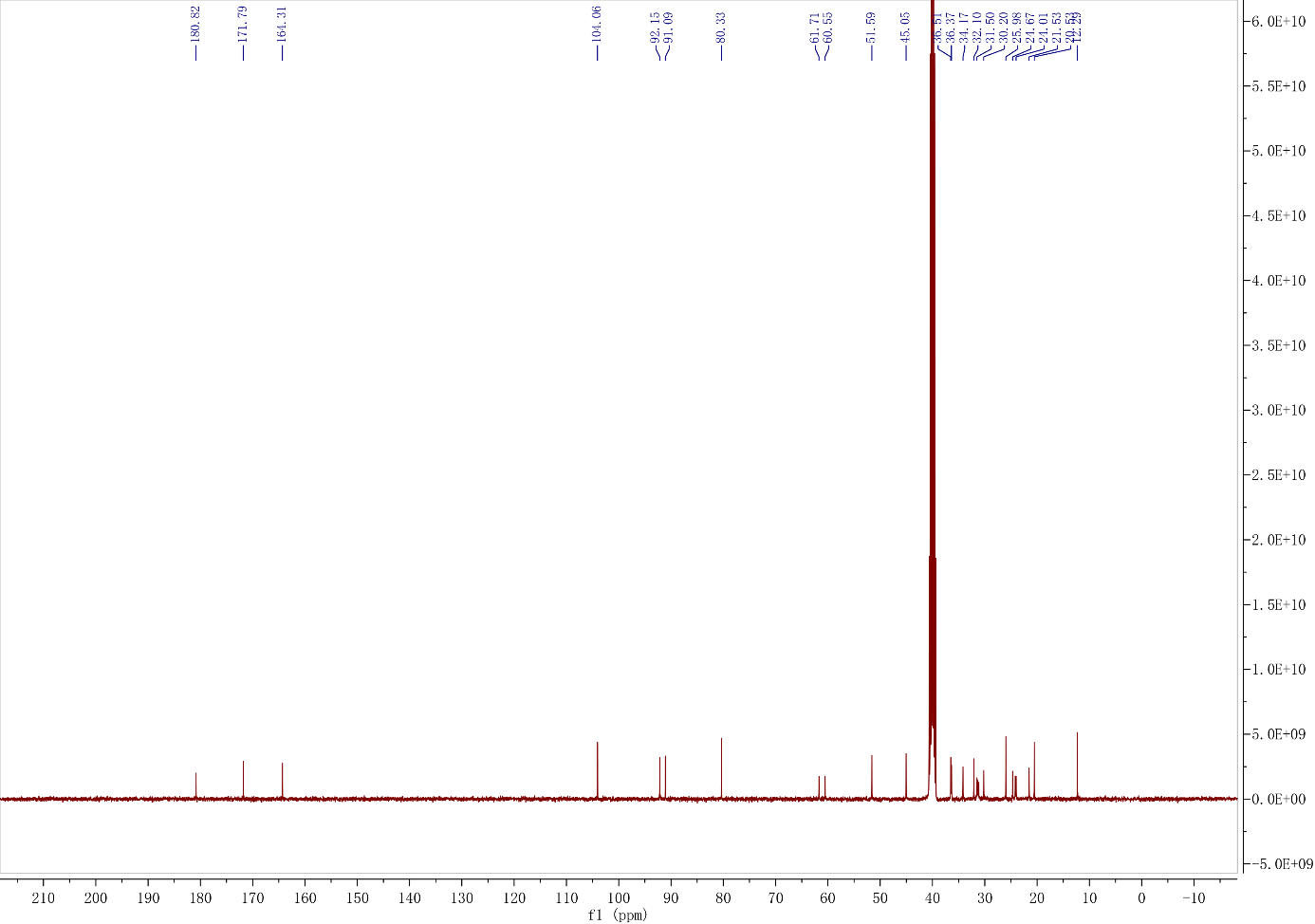


# Figure S2. ^13^C spectrum of OART.


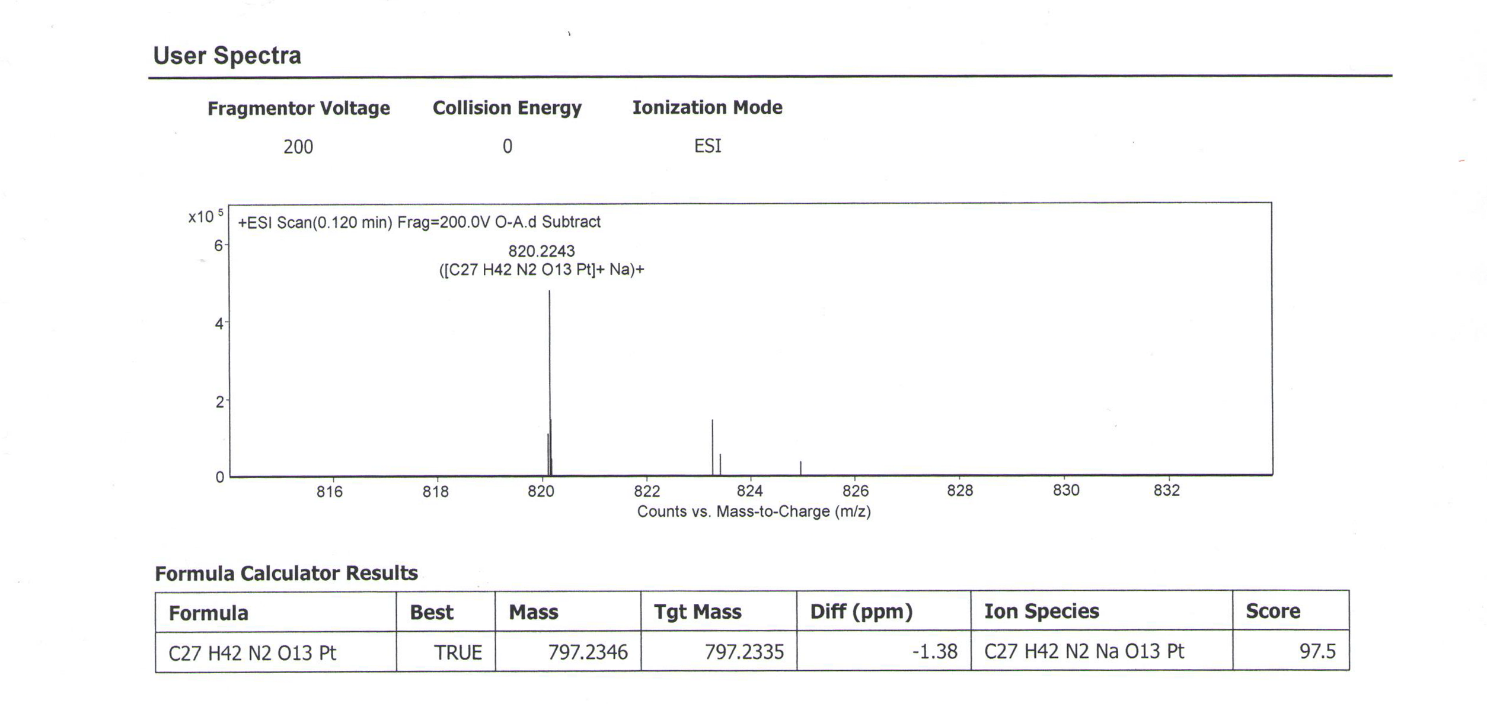


# Figure S3. HRMS of OART.


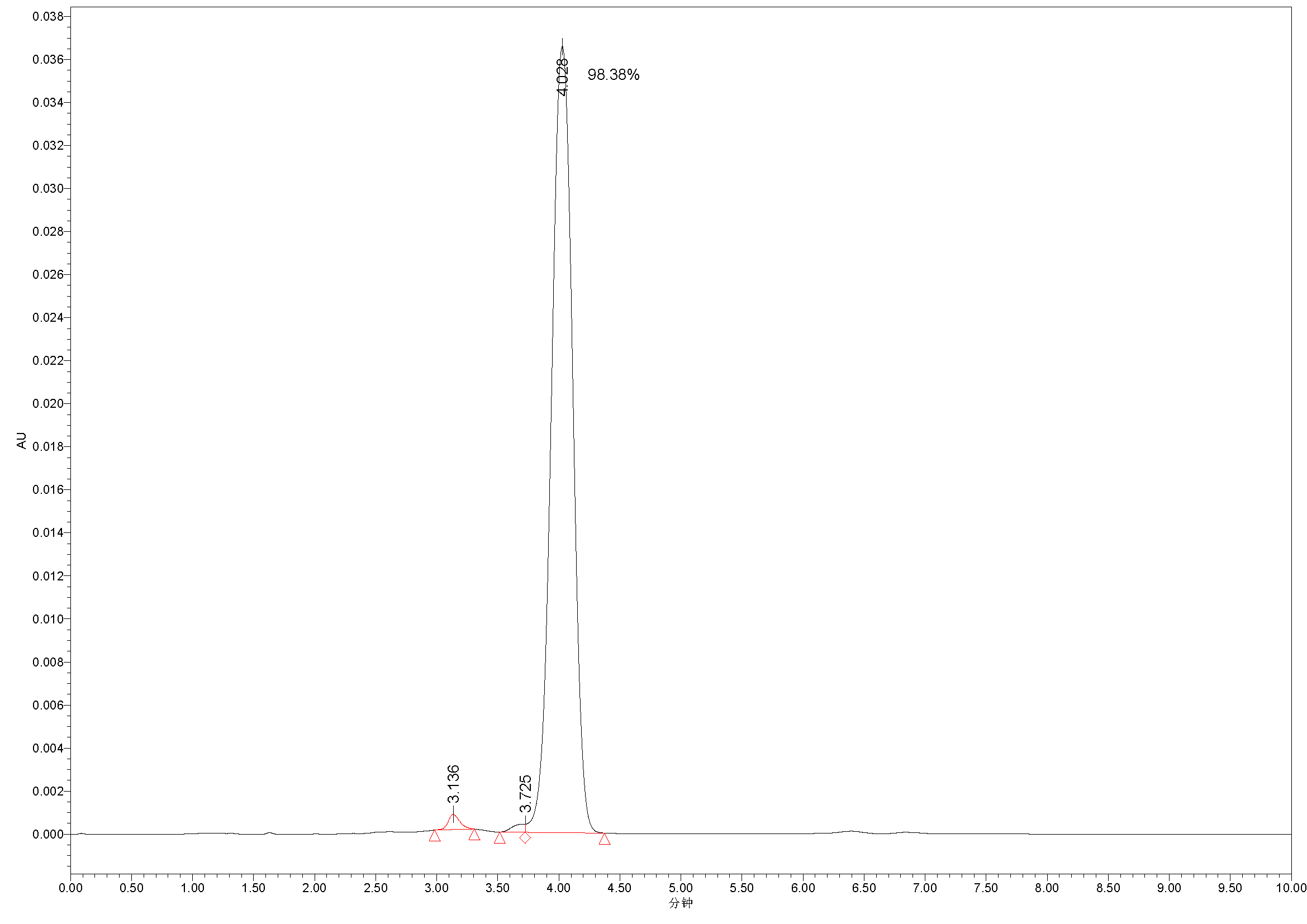


# Figure S4. HPLC of OART.





# Figure S5. Time dependent morphological observation on 4T1 cells after treatment with Oxa, ART, and OART.





# Figure S6. OART inhibit cell migration in 24 h. (n=3)


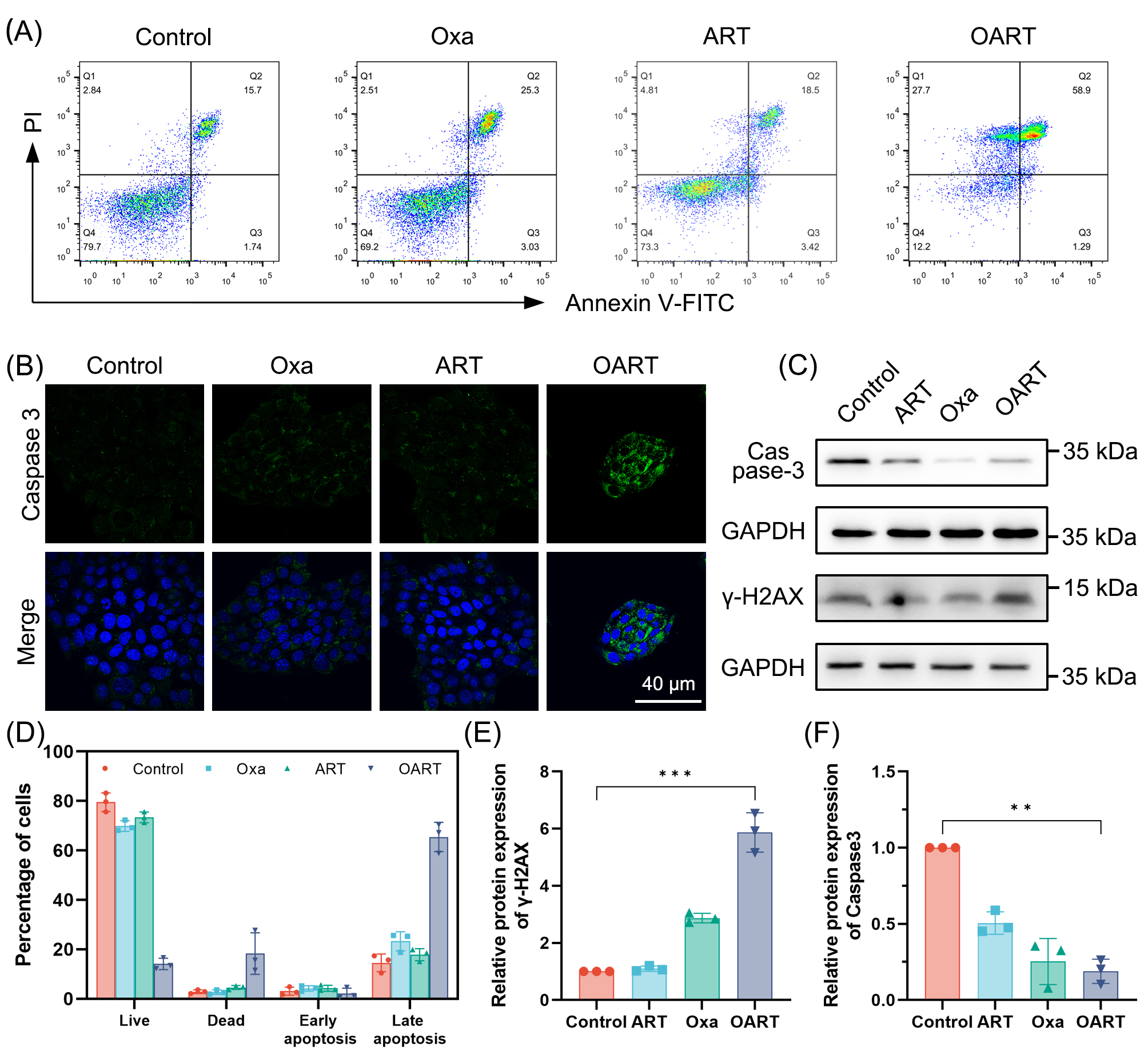


# Figure S7. OART's ability to induce apoptosis and DNA damage. (A and D) Flow experiments and statistical analysis of apoptosis. (n=3) (B) Immunofluorescence staining of cellular activity Caspase-3. Scale bars = 40 μm. (C and E-F) Western blot images and statistical analysis of Caspase-3 and DNA damage marker γ-H2AX. (n=3) Data are expressed as mean ± SD. ***p* <0.01, ****p* <0.001.





# Figure S8. Ferroptosis inhibitor rescue experiment. (A-B) Cellular activity of DFO (A) and fer-1 (B) coincubation with different compounds. Data are expressed as mean ± SD. (n=3) ***p* <0.01, *****p* <0.0001.


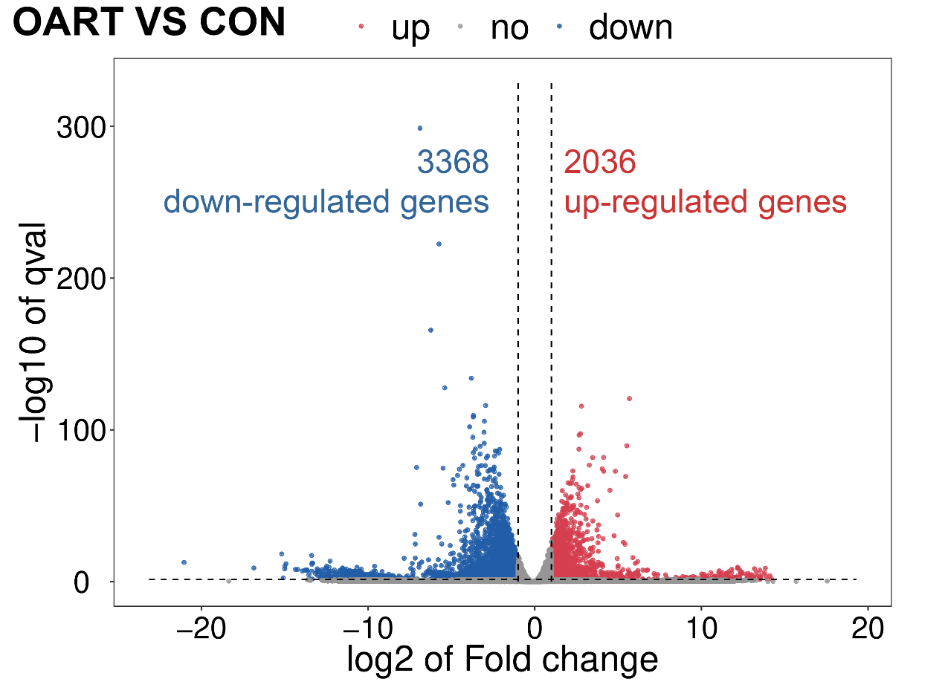


# Figure S9. OART treatment causes multiple genes changed in 4T1 cells.





# Figure S10. OART downregulates GPX4 expression in tumor tissue. (A-B) Western blot images and statistical analysis of GPX4. (n=3) (C) Immunohistochemical images of GPX4. Scale bars = 50 μm. *****p* <0.0001





# Figure S11. Blood component analysis in mice treated with indicated drugs. (n=3)





# Figure S12. Body weight changes and survival in mice with lung metastases. (n=5) (A) Body weight. (B) Survival curve. (C) The number of lung nodules. ****p* <0.001, *****p* <0.0001.


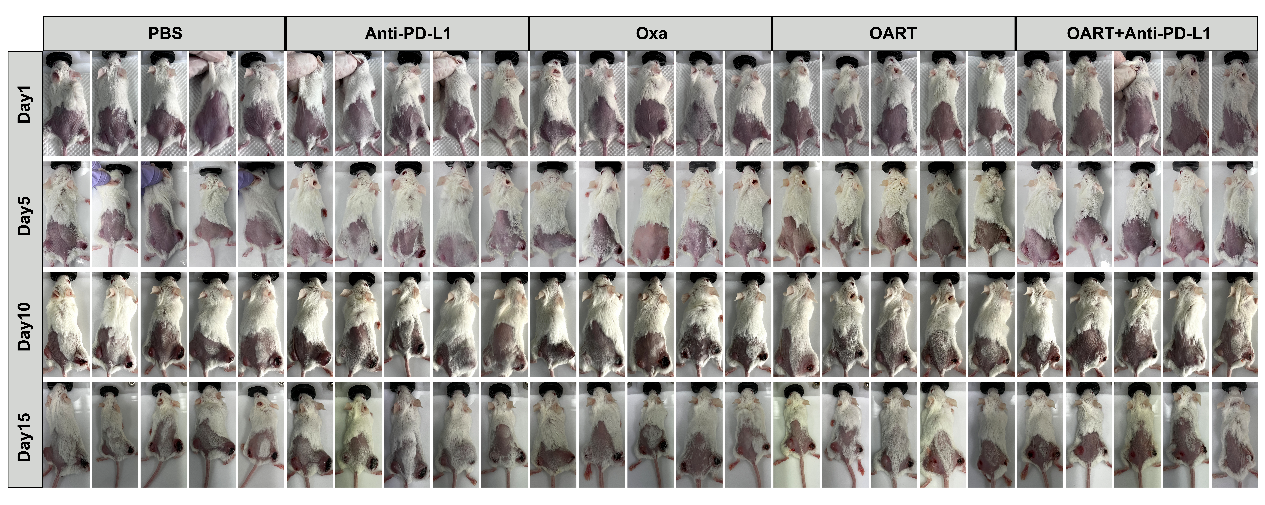


# Figure S13. Image of bilateral tumors in mice with indicated drugs. (n=5)


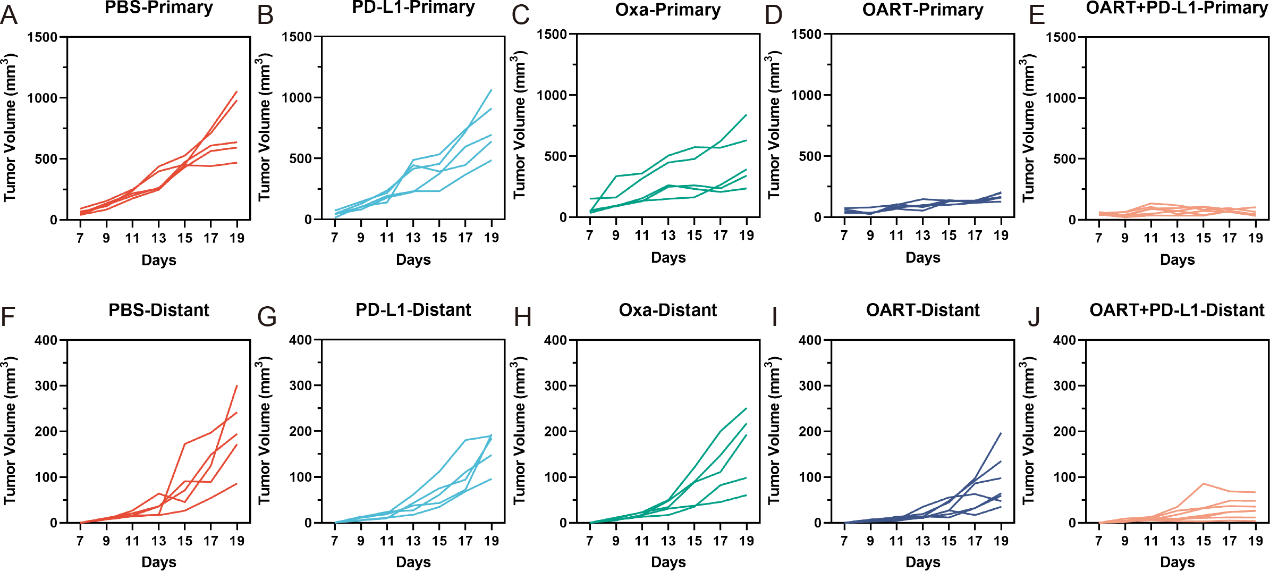


# Figure S14. Growth curves of Image of bilateral tumors in mice with indicated drugs. (n=5)
